# Is Dental Anxiety Associated with Oral Health-Related Quality of Life? Assessment of Statistical Significance and Clinical Meaningfulness in a Sample of Russian Medical and Dental Students

**DOI:** 10.3390/dj11110260

**Published:** 2023-11-07

**Authors:** Christine Nordbø Heyeraas, Silje Nyborg Jensen, Vilde Bjørkli Stabell, Jan-Are K. Johnsen, Sergei N. Drachev

**Affiliations:** Department of Clinical Dentistry, Faculty of Health Sciences, UiT The Arctic University of Norway, N-9037 Tromsø, Norway

**Keywords:** medical students, dental students, North-West Russia, dental anxiety, oral health-related quality of life, minimal clinically important difference

## Abstract

Information about oral health-related quality of life (OHRQoL) and dental anxiety (DA) in Russian young adults is scarce. We investigated how DA is associated with OHRQoL in a group of medical and dental students in North-West Russia. The study had a cross-sectional design and included 807 students aged 18–25 years who attended the Northern State Medical University in Arkhangelsk. OHRQoL and DA were measured by the Oral Health Impact Profile (OHIP-14) and Corah’s Dental Anxiety Scale (DAS), respectively. A questionnaire collected information on socio-demographics and self-reported oral health (OH) characteristics. A dental examination was executed to assess dental caries and oral hygiene. We observed differences in the OHIP-14 scores between dentally anxious and non-anxious students: unadjusted incidence rate ratio [IRR] = 1.65, 95% confidence interval [CI]: 1.29–2.12; after adjustment for socio-demographics and clinically assessed OH: IRR = 1.58, 95% CI: 1.23–2.02; after adjustment for socio-demographics, clinically assessed OH, and self-reported OH characteristics: IRR = 1.27, 95% CI: 0.99–1.63. The differences between estimated marginal means for the DAS categories in the models were 2.92, 2.51, and 1.24, respectively. Minimal clinically important differences of OHIP-14 fell between 1.68 and 2.51. We found a negative statistical association between DA and OHRQoL in our study sample, but after adjustment for potential confounders, the association lost its clinical importance.

## 1. Introduction

The World Health Organization (WHO) defines health as “a state of complete physical, mental and social well-being and not merely the absence of disease or infirmity” [1]. Oral health (OH) is an integral part of general health, and oral diseases affect millions of people worldwide causing pain, discomfort, and social and psychological problems [2]. Along with clinical indicators determined by dental professionals, OH assessment should also include a measure of patients’ subjective state of OH. To identify how oral diseases impact a person’s physical, emotional, and social well-being, researchers use the concept of OH-related quality of life (OHRQoL) [3,4,5]. Since the 1980s, a set of instruments has been designed to measure OHRQoL. One of the most widely used tools is the OH Impact Profile (OHIP) that comprises 49 statements (OHIP-49) on negative impacts on OHRQoL [6]. To reduce the number of items, a short version of OHIP that includes 14 statements (OHIP-14) has been proposed [7], and many studies have demonstrated its acceptable psychometric properties [4].

OHRQoL is one of several patient-based outcome measures that are often difficult to interpret because they do not have a meaningful benchmark [8]. Indeed, the interpretation should not be based solely on reporting aggregate scores and detecting statistically significant differences between groups at one point of time or over time [8]. The minimal clinically important difference (MCID), which is defined as “the minimal change in score considered relevant by patients and physicians” [9], should also be reported. To calculate MCID, there are two well-defined methods, distribution-based and anchor-based [8,9,10]. Distribution-based, or internally referenced, methods focus on the distribution of observed scores in the sample and include the calculation of effect size, standard error of measurement, or multiples of standard deviation [8,9,10]. Anchor-based, or externally referenced, methods use clinical (i.e., laboratory data, physiological criteria, or clinician ratings) or patient-based (i.e., subjective global transition scales) indicators to compare outcome scores [8,9,10]. Given that there is no clear consensus on which methods are more suitable to determine MCIDs, it is strongly recommended to use both multiple independent anchors and distribution-based methods [9,10]. To the best of our knowledge, there has been little information on MCIDs for OHIP-14 scores. A study conducted among low-income and institutionalized elderly in the City of Toronto reported that the MCID for OHIP-14 scores, with a 5-point response scale, is equal to 5 [11].

Many studies investigated factors associated with OHRQoL [12,13,14,15,16,17,18,19,20,21,22,23,24]. Reported predictors for low OHRQoL have included low socioeconomic position [13,14,18,19,21,22], poor self-reported OH characteristics [14,23], clinical OH status [12,13], and OH behavior [19,21]. In addition, dental anxiety (DA) was negatively associated with OHRQoL [12,13,14,15,16,17,18,19,20,24]. In different countries and cultures, the prevalence of DA was found to vary from 2% to 30% [25,26]. Several studies have shown that DA is more common in younger age groups [27,28]. Moreover, a longitudinal study conducted in New Zealand reported an increase in DA among young adults aged 18–26 years over eight years [29]. Nevertheless, we did not find studies that investigated the association between OHRQoL and DA from both statistical and clinical points of view.

Although poor OH was reported in Russian young adults [30], information on their OHRQoL and DA is scarce. In 2015–2016, a study among Russian medical and dental students aged 18–25 years was conducted to investigate OHRQoL and DA and its associated factors [31,32]. More than half of the students (53.6%) had low OHRQoL. Socio-demographics, poor self-reported OH characteristics, and high dental caries experience were reported to be associated with low OHRQoL [31]. Only statistical significance was determined, and no clinical significance of the factors in relation to OHRQoL was assessed. The prevalence of DA was reported to be 2.2% and 13.7% in the groups of dental and medical students, respectively [32]. Nevertheless, the association between OHRQoL and DA is still unknown in the study sample. The study aims to investigate how DA is associated with OHRQoL in the Russian medical and dental students, and whether this association is clinically meaningful in crude analysis and after adjustment for socio-demographics, clinically assessed OH, and self-reported OH characteristics.

## 2. Materials and Methods

### 2.1. Study Design and Population

The study had cross-sectional design and was based on data collected at the Northern State Medical University (NSMU), Arkhangelsk, North-West Russia. Altogether, ~3900 students mainly from the European North-West of Russia attended the NSMU. Undergraduate Russian students from two faculties, medical (*n* = 1482) and dental (*n* = 524), were invited to participate in the study in the 2015–2016 academic year. 

### 2.2. Sampling

**Figure 1:**
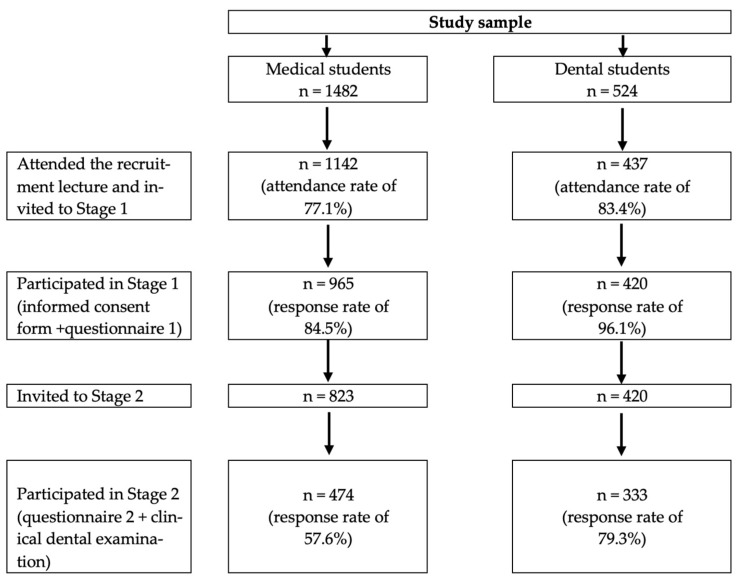
Flow chart of the study sample.

Sampling was employed in two stages. At Stage 1, one of the researchers (Sergei N. Drachev) visited a randomly selected curriculum classroom lecture that was given to medical and dental students of each year of education. All students who attended the recruitment lecture were informed about the study and invited to participate. After signing the informed consent form, the students who agreed to participate completed a structured, self-administered questionnaire in Russian. At Stage 2, a sample of medical students and all dental students who participated in Stage 1 were asked to complete a second structured, self-administered questionnaire in Russian. In addition, these students were clinically examined at the NSMU Dental Clinic applying the following inclusion criteria: Russian nationality, age between 18 and 25 years, absence of fixed orthodontics bands, and non-pregnant women. More details related to sampling and sample size calculation have been published elsewhere [33]. The brief flow chart of the study sample is presented in Figure 1.

### 2.3. Oral Health-Related Quality of Life (Outcome Variable)

The Russian version of the OHIP-14, previously validated and reported elsewhere [34], was included in the Stage 2 questionnaire and used to assess OHRQoL. The OHIP-14 questionnaire includes a total of 14 items within seven dimensions that reflect negative impacts on OHRQoL: functional limitation, physical pain, psychological discomfort, physical disability, psychological disability, social disability, and handicap. Participants were asked to indicate the frequency with which they experienced negative conditions related to their teeth, mouth, or dentures in the last year. They chose one response from the following response options: (0) never, (1) hardly ever, (2) occasionally, (3) fairly often, and (4) very often. In addition, the study participants could select the response option “I do not know” for each item. If a student chose that response for at least one item, the student was excluded from the statistical analysis. The total OHIP-14 score that was calculated as the sum of all OHIP-14 items ranged from 0 to 56 points. A higher total OHIP-14 score reflected a lower OHRQoL. The psychometric properties of the OHIP-14 have been assessed previously, with both construct validity and internal consistency reported to be satisfactory [31]. 

### 2.4. Dental Anxiety (Exposure Variable)

To measure DA, Corah’s Dental Anxiety Scale (DAS) [35] was included in the Stage 2 questionnaire. The forward translation of the original version of DAS, which contains four items in English, into Russian was conducted by two bilingual persons independently. Then, the backward translation into English was performed by another two translators. The original and back-translated versions were compared to ensure the equivalence of the scale. Moreover, a pilot study that included the DAS questionnaire was carried out among students of the target age group who were not involved in the main study. Participants were asked to answer the DAS items on a 5-level scale. These scores were summed, and the total DAS score ranged from 4 to 20. In accordance with previously published studies, a DAS score ≥ 13 was defined as indicating high DA [32,36]. The psychometric properties of the DAS have been reported elsewhere [32]. 

### 2.5. Potential Confounders

Information on socio-demographics and self-reported OH characteristics was included in the Stage 1 questionnaire. Sex, age group (18–20/21–25 years), faculty (dental/medical), and place of childhood residence (rural/urban) were considered socio-demographic variables. Self-reported OH characteristics included self-assessed dental aesthetic, dichotomized as “good” (i.e., excellent/very good/good) and “poor” (i.e., fair/poor), and satisfaction with mouth and teeth with three response options: (1) yes, (2) no, and (3) difficult to answer. Information on dental caries experience, expressed as the sum of decayed (D), missing (M), and filled (F) teeth (T) index, and the Simplified Oral Hygiene Index (OHI-S) of Green and Vermillion (1964) was gathered within a dental examination at the Dental Clinic of NSMU. All permanent teeth, excluding third molars, were visually and tactilely examined to calculate the DMFT index. The OHI-S index was recorded as the sum of the average individual debris and calculus scores [37]. One calibrated dentist (Sergei N. Drachev) performed all clinical dental examinations in line with the WHO recommendations [38], and the level of intra-examiner agreement was found to be satisfactory [33]. 

### 2.6. Assessment of Minimal Clinically Important Difference in OHIP-14 Score

To calculate the MCID of the OHIP-14 score, both anchor-based and distribution-based methods were applied. For the anchor-based method, we used one global question asking how students rate their OH. The response options included (1) excellent; (2) very good; (3) good; (4) fair; and (5) poor. In addition, the students could choose the response option “Difficult to answer” for the question. If a student selected that response, he/she was excluded from the analysis. The Spearman rank correlation between self-assessed OH and OHRQoL (OHIP-14 score) was 0.334 (*p* < 0.001), that is, it was greater than the value of 0.3 that allowed for the inclusion of the variable “self-assessed OH” as an anchor [10]. For the distribution-based method, we calculated ½ standard deviation (SD) and the standard error of measurement (SEM) [10], defined as

$$\mathrm{SEM}=\mathrm{SD}*\sqrt{1-\mathrm{reliability}},$$

where reliability is Cronbach’s alpha for OHIP-14 equal to 0.85 [31].

### 2.7. Statistical Analysis

IBM SPSS Statistics for Macintosh, version 28.0 (IBM Corp., Armonk, NY, USA) was used for statistical analysis. Descriptive statistics were applied to describe characteristics of the sample. The Spearman rank correlation coefficient between OHIP-14 and DAS scores was calculated. Given the distribution of the dependent variable (OHIP-14 score) [31], negative binomial regression with robust estimates was used to assess the association between DA and OHRQoL. At the first stage, simple regression was run to find the crude association between OHRQoL (OHIP-14 score) and DAS categories. At the second stage, socio-demographic factors, clinically assessed OH, and self-reported OH characteristics were sequentially entered in the multivariable regression model to find the adjusted association between the outcome and exposure. The final model was checked for multicollinearity, and there was no violation of the assumption. Given a non-significant interaction between the faculty and DA in relation to the OHIP-14 score, the study results were presented for the entire study group of medical and dental students. The significance level (p) was set at 5% for all statistical tests. 

### 2.8. Ethical Considerations

Ethical approval for the research project was obtained from both the Ethical Committee of the NSMU, Russia, and the Regional Ethical Committee of Norway. Participation in the study was based on signed written informed consent.

## 3. Results

A total of 807 students of the NSMU agreed to participate in the study. Three-quarters of the students were women; 60% were aged 18–20 years; and 70.9% reported an urban place of childhood residence. Almost 60% of the students reported good dental aesthetic, 43% had good self-assessed OH, and 42% were satisfied with their mouth and teeth. The overall mean DMFT and OHI-S indexes were 7.55 and 1.13, respectively. The DAS score ≥ 13 was found in 9.4% of medical and dental students (Table 1). 

Of the 807 medical and dental students, 729 students answered all items of the OHIP-14 questionnaire and were included in the analysis. The OHIP-14 score ranged from 0 to 34 with a mean value of 4.72 (SD = 5.02).

DAS scores were correlated with OHIP-14 scores (Spearman rank correlation coefficient = 0.162, *p* < 0.001). There were statistically significant differences in the OHIP-14 scores between dentally anxious and non-anxious students in the crude analysis and in the analysis adjusted for socio-demographic factors and clinically assessed OH (Table 2, Models 1–3). For example, students who reported a DAS score ≥ 13 had an adjusted OHIP-14 score that was 1.58 (95% CI: 1.23–2.02) times higher than that found in those with a DAS score < 13 (Table 2, Model 3). We found marginally statistically significant differences in OHIP-14 score between students with and without DA adjusted for socio-demographics, clinically assessed OH, and self-reported OH characteristics (Table 2, Model 4). The differences between estimated marginal means for DAS categories in the models varied from 1.24 to 2.92 (Table 2).

The MCID calculations based on the anchor-based approach are presented in Table 3. A value of 1.68 was found to be the MCID. The MCIDs, calculated as ½ SD and SEM, were 2.51 and 1.94, respectively. Considering both the anchor-based and distribution-based methods, one may assume that the MCID for OHIP-14 scores was within the interval from 1.68 to 2.51.

## 4. Discussion

Our study found a negative association between DA and OHRQoL in the sample of Russian medical and dental undergraduate students. The statistical association was clinically meaningful both in crude analysis and after adjustment for socio-demographic factors and clinically assessed OH. Nevertheless, after additional adjustment for self-reported OH characteristics, it lost its clinical importance, although marginally statistically significant differences between OHIP-14 scores in dentally anxious and non-anxious students were still observed. 

To our knowledge, the present study is the first attempt to evaluate the association between OHRQoL and DA in Russian adults aged 18–25 years both from statistical and clinical points of view. To measure OHRQoL and DA, we used OHIP-14 and DAS scales, which are instruments with a good internal consistency and good/sufficient face and construct validity [31,32]. To assess OH, all study participants were clinically examined in accordance with the recommendations of the WHO, and the level of intra-examiner agreement was satisfactory [32]. Nevertheless, the study does have a set of limitations. Firstly, only medical and dental students from one Russian University were invited to participate in the study; therefore, the extrapolation of our findings to other groups of Russian students or Russian young adults at large may be questioned. Secondly, although we applied both anchor-based and distribution-based methods to evaluate clinical meaningfulness of OHIP-14 scores, only one global question (self-assessed OH) was employed as an anchor, whereas a multi-item instrument is considered to be a more valid and reliable measure [39]. Moreover, to find the MCID, we calculated the mean of mean differences in OHIP-14 scores between the adjacent categories of self-assessed OH, which varied from 0.91 to 2.39. Despite the recommendations for using the MCID calculations, more evidence is needed to use this approach [8]. In addition, the distribution-based methods applied in the present study might evaluate minimal detectable changes in OHIP-14 scores rather than MCIDs [39]. Thirdly, given that the present study has a cross-sectional design, we cannot conclude on a causal relationship between OHRQoL and DA and investigate a trend in OHIP-14 scores over time. Longitudinal studies should be designed to answer the following question: Does high DA cause low OHRQoL, or does low OHRQoL cause high DA? Fourthly, we did not assess test–retest reliability both for OHIP-14 scores and DAS scores. Finally, dental caries were detected visually and tactilely only, without taking radiographs, which could lead to an under-reporting of dental caries. 

We did not find other studies that have investigated a relationship between DA and OHRQoL in medical and dental undergraduate students. Nevertheless, a set of studies have explored the association in samples from general populations. A large British study, which included 1800 people aged 16 years and older from England, Wales, and Scotland, reported a correlation coefficient of 0.14 between DAS and OHRQoL scores that is in line with our finding (0.16) [20]. A German study conducted among adult patients with DA found a correlation coefficient of 0.25 (95% CI: 0.10–0.38) between DAS and OHIP-14 summary scores that corresponds to our study results [17]. It was suggested the statistically significant weak correlation between DA and OHRQoL may not reflect a clinical relevance [40]. In the British study, DA remained a statistically significant predictor of low OHRQoL after adjustment for socio-demographics (sex, age, and social class) and self-reported OH [20], which is in line with our findings. An Indian cross-sectional study among 1235 participants aged 15–54 years reported a negative association between DA and OHRQoL, controlling for socio-demographics, socioeconomics, number of teeth, and dental attendance [24]. In a Swedish cross-sectional national survey, high DA was independently related to low OHRQoL adjusted for irregular dental care, smoking, age, and gender [15]. In our study, we also found a similar statistical association between DA and OHRQoL; but having accounted for self-reported OH characteristics, the differences in OHIP-14 scores between dentally anxious and non-anxious students became clinically nonmeaningful. The reason for this might be that DA and OHRQoL, and their measurements, may reflect similar psychological constructs or states [20]. Indeed, the psychosocial impact of DA was reported to have five main impacts on daily living: physiological, cognitive, behavioral, health-related, and social [41]. These impacts may be correlated with the OHIP-14 dimensions of a negative impact on OHRQoL. Moreover, individuals with high DA who visit the dentist irregularly have poor OH [42] that may lead to a lower OHRQoL. Taking into account the above-mentioned suggestions, one may speculate that DA is highly likely to be associated with OHRQoL, and this association was reported in other studies [20,24]. In our study, similar findings were observed in the crude analysis and in the analysis adjusted for socio-demographic factors and clinically assessed OH. Nevertheless, when adjusting for the self-reported OH characteristics, the independent impact of DA on OHRQoL became non-significant from the clinical point of view, although it still remained marginally statistically significant. This finding may be explained by the fact that in our study sample, dissatisfaction with mouth and teeth and poor self-assessed dental aesthetic were reported to be the strongest factors associated with low OHRQoL [31]. These self-reported factors might best reflect physical pain and psychological discomfort, which were found to be the biggest drivers of low OHRQoL in our students [31]. This corresponds to findings from other studies, where self-reported OH conditions impacted OHRQoL to a greater extent than clinically diagnosed oral diseases [12]. 

In our study sample of Russian undergraduate students aged 18–25 years, we found that the MCID for OHIP-14 scores likely falls between the values of 1.68 and 2.51 points. The comparisons with other studies are complicated because information on the MCID of OHIP-14 scores is scarce. We found only one study that assessed the responsiveness of the OHRQoL measure in 116 patients who completed the OHIP-14 questionnaire prior to treatment and one month after the treatment. In contrast with our findings, an MCID of 5 for OHIP-14 scores was observed [11]. On one hand, the researchers reported that a large sample size is needed to validate the results [11]. On the other hand, the study included elderly adults aged 59–88 years, whereas only young adults aged 18–25 years participated in our study. We cannot exclude that the perception of OH problems and their impact on OHRQoL may be different in elderly and in young adults. Indeed, a deleterious effect of oral diseases on subjective OH was suggested to be higher at younger ages [43] which, to some extent, may explain a smaller MCID in our study. 

## 5. Conclusions

DA is negatively associated with OHRQoL in Russian medical and dental undergraduate students aged 18–25 years. The statistical association was clinically meaningful in crude analysis and after adjustment for socio-demographic factors and clinically assessed OH. Nevertheless, after additional adjustment for self-assessed dental aesthetic and satisfaction with mouth and teeth, it lost the clinical importance, despite marginally statistically significant differences between OHIP-14 scores in dentally anxious and non-anxious students. We found that the MCID for OHIP-14 scores was between the values of 1.68 and 2.51 points. More studies with representative samples of young adults are warranted to validate our findings. 

## Figures and Tables

**Table 1:** Characteristics of the sample of Russian medical and dental students, *n* = 807.

Variable	Categories	*n* (%)
Age group, years	18–20	483 (59.9)
	21–25	324 (40.1)
Sex	Male	208 (25.8)
	Female	599 (74.2)
Faculty	Medical	474 (58.7)
	Dental	333 (41.3)
Place of childhood residence	Urban	572 (70.9)
	Rural	232 (28.7)
	Missing	3 (0.4)
Self-assessed dental aesthetic	Good	482 (59.7)
	Poor	315 (39.0)
	Missing	10 (1.2)
Satisfaction with mouth and teeth	Yes	341 (42.3)
	No	345 (42.8)
	Difficult to answer	119 (14.7)
	Missing	2 (0.2)
DAS score	Less than 13	728 (90.2)
	13 and more	76 (9.4)
	Missing	3 (0.4)
DMFT, mean (SD)		7.55 (4.40)
OHI-S, mean (SD)		1.13 (0.52)
Self-assessed oral health	Excellent	44 (5.5)
	Very good	106 (13.1)
	Good	344 (42.6)
	Fair	251 (31.1)
	Poor	30 (3.7)
	Difficult to answer	32 (4.0)

*Abbreviations:* DAS, Dental Anxiety Scale; DMFT, decayed, missing, and filled teeth; OHI-S, Simplified Oral Hygiene Index; SD, standard deviation.

**Table 2:** Statistical association between exposure (dental anxiety) and outcome (oral health-related quality of life) in the study sample: results from simple and multivariable negative binomial regression.

Model *	Dental Anxiety	IRR (95% CI)	*p* Value	Estimated Marginal Means (95% CI)	Difference between Estimated Marginal Means
1	DAS score		<0.001		2.92
	less than 13	Reference		4.48 (4.13–4.85)	
	13 and more	1.65 (1.29–2.12)		7.40 (5.84–9.38)	
2	DAS score		<0.001		2.67
	less than 13	Reference		4.37 (3.96–4.83)	
	13 and more	1.61 (1.25–2.08)		7.04 (5.45–9.10)	
3	DAS score		<0.001		2.51
	less than 13	Reference		4.37 (3.96–4.82)	
	13 and more	1.58 (1.23–2.02)		6.88 (5.32–8.88)	
4	DAS score		0.055		1.24
	less than 13	Reference		4.50 (4.07–4.98)	
	13 and more	1.27 (0.99–1.63)		5.74 (4.45–7.39)	

*Abbreviations:* IRR, incidence rate ratio; CI, confidence interval; DAS, Dental Anxiety Scale. * 1—model with unadjusted estimates; 2—model with estimates adjusted for socio-demographics (age group, sex, faculty, place of childhood residence); 3—model with estimates adjusted for socio-demographics and clinically assessed OH (DMFT index and OHI-S); 4—model with estimates adjusted for socio-demographics, clinically assessed OH, and self-reported OH characteristics (self-assessed dental aesthetic, satisfaction with mouth and teeth).

**Table 3:** Anchor-based approach for assessment of minimal clinically important differences in OHIP-14 scores in the study sample.

Self-Assessed Oral Health	Mean OHIP-14 Score	Difference between the Adjacent Categories	Mean of the Differences (MCID Estimate)
Excellent	1.77	1.32 (=3.09–1.77)	(1.32 + 0.91 + 2.39 + 2.09)/4 = 1.68
Very good	3.09	0.91 (=4.00–3.09)	
Good	4.00	2.39 (=6.39–4.00)	
Fair	6.39	2.09 (=8.48–6.39)	
Poor	8.48		

*Abbreviations:* OHIP, Oral Health Impact Profile; MCID, minimal clinically important differences.

## Data Availability

Not applicable.
